# Loss of Urokinase Receptor Sensitizes Cells to DNA Damage and Delays DNA Repair

**DOI:** 10.1371/journal.pone.0101529

**Published:** 2014-07-02

**Authors:** Pavan B. Narayanaswamy, Mahshid Hodjat, Hermann Haller, Inna Dumler, Yulia Kiyan

**Affiliations:** Nephrology Department, Hannover Medical School, Hannover, Germany; The University of Hong Kong, Hong Kong

## Abstract

DNA damage induced by numerous exogenous or endogenous factors may have irreversible consequences on the cell leading to cell cycle arrest, senescence and cell death. The DNA damage response (DDR) is powerful signaling machinery triggered in response to DNA damage, to provide DNA damage recognition, signaling and repair. Most anticancer drugs induce DNA damage, and DNA repair in turn attenuates therapeutic efficiency of those drugs. Approaches delaying DNA repair are often used to increase efficiency of treatment. Recent data show that ubiquitin-proteasome system is essential for signaling and repair of DNA damage. However, mechanisms providing regulation of proteasome intracellular localization, activity, and recruitment to DNA damage sites are elusive. Even less investigated are the roles of extranuclear signaling proteins in these processes. In this study, we report the involvement of the serine protease urokinase-type plasminogen activator receptor (uPAR) in DDR-associated regulation of proteasome. We show that in vascular smooth muscle cells (VSMC) uPAR activates DNA single strand break repair signaling pathway. We provide evidence that uPAR is essential for functional assembly of the 26S proteasome. We further demonstrate that uPAR mediates DNA damage-induced phosphorylation, nuclear import, and recruitment of the regulatory subunit PSMD6 to proteasome. We found that deficiency of uPAR and PSMD6 delays DNA repair and leads to decreased cell survival. These data may offer new therapeutic approaches for diseases such as cancer, cardiovascular and neurodegenerative disorders.

## Introduction

Genomic instability resulting from damaged DNA causes many diseases such as cancer, cardiovascular and neurodegenerative disorders, immune deficiencies and metabolic syndrome [1]. Both exogenous factors like ultraviolet light, ionizing radiation, environmental chemicals and endogenous sources like reactive oxygen species can induce DNA damage. Moreover, many drugs used to treat cancer, psoriasis, and some other disorders have been identified as DNA-damaging agents [2], [3]. To combat DNA damage, cells evolved the DNA damage response (DDR), which represents highly coordinated signaling mechanisms aiming at recognizing DNA lesions, signaling their appearance, and providing efficient repair. Deficiency and failures in DDR mechanisms lead to increased cell sensitivity to DNA-damaging factors. Therapeutic inhibition of DNA repair increases efficiency of DNA damage-inducing drugs [4]. This explains the research interest in revealing comprehensive mechanisms of DDR signaling and DNA repair.

Recent studies have elucidated that the ubiquitin-dependent proteasome degradation system (UPS) is involved in coordination of DDR after DNA damage [5]. The 26S proteasome consists of the 19S regulatory particles and the 20S catalytic core particle with protease activity. ATPase subunits of 19S regulatory particle form the base and non-ATPase regulatory subunits form the lid complex of 19S. Molecular organization and assembly of 26S proteasome subunits are crucial for regulation of proteasome activity [6]. It was reported that inhibition of proteasome activity impairs DNA repair and DNA damage-induced apoptosis in cancer cells [7]. Other studies demonstrated that proteasome plays rather a negative role in DDR [8]. Evidence suggested that proteasome might regulate DDR either indirectly via availability of ubiquitin pool or directly by deubiquitinating and degrading DDR proteins. Further research revealed the proteolysis-independent role of 19S regulatory particle [9] or separate 19S subunits [10]. How functional properties and assembly of 26S proteasome are regulated and orchestrated upon DDR remains, however, poorly explored.

DDR pathways have been studied primarily on proliferating cells relevant to cancer therapy. Mechanisms of DDR in post-mitotic terminally differentiated cells might, however, differ significantly. Thus, main DNA repair mechanisms are downregulated in postmitotic cells leading to accumulation of DNA damage. Nevertheless, terminally differentiated cells are more resistant to genotoxic stressors. Vascular smooth muscle cells (VSMC) are not terminally differentiated and are capable of dedifferentiation to acquire proliferating synthetic phenotype. VSMC proliferation plays an important role in the physiological process of repair of vascular injury as well as in pathological vascular remodeling associated with diseases such as atherosclerosis and post-angioplasty restenosis. Extensive evidence documents DNA damage in atherosclerosis. Furthermore, large cohort studies confirmed significant increase of cardiovascular events after cytotoxic chemotherapy [11]. Our recent data and reports of others demonstrated cytotoxic action of anti-cancer drug doxorubicin (Dox) on VSMC. We showed that proteasome activity is implicated in developing VSMC senescence after Dox treatment and that the proteasome activity is in turn regulated by the multifunctional urokinase (uPA)/urokinase receptor (uPAR) system [12], [13].

uPA/uPAR play a central role in molecular events coordinating functional behavior and cell fate in health and disease [14], [15]. Though uPA/uPAR interference with DDR has not been proved experimentally, several clues from different studies suggest that uPA/uPAR might also be involved in at least some pathways triggered by DNA damage. Thus, in different cell types this system regulates main cellular functions related to DDR, such as proliferation, cell cycle, senescence, and apoptosis [16], [17]. We and others have demonstrated recently that uPAR possesses transcriptional activity and may undergo nuclear translocation and regulate cellular events at nuclear level that further strengthens the implication of uPAR in DDR-related processes [12], [18].

In the present study, we demonstrate that uPAR serves as an active participant in DDR signaling events. uPAR-deficient cells are sensitized to DNA damage and reveal decreased survival as a result of impaired DNA repair. We further show that underlying molecular mechanisms involve uPAR-mediated regulation of the 26S proteasome assembly and activity via phosphorylation and nuclear import of the 19S regulatory subunit PSMD6.

## Results

### uPAR is required for 26S proteasome assembly

We reported recently a new role for uPAR in Dox-induced senescence in VSMC via UPS regulation. Dox as an intercalating agent and topoisomerase II inhibitor induces DNA double strand breaks (DSB) and DDR signaling. In order to clarify how uPAR interferes with Dox-induced proteasome activity, we now studied proteasome assembly using mass spectrometry. As shown in Table 1, recruitment of several regulatory 19S lid subunits, in particular PSMD6, PSMD7, and PSMD13 to proteasome complex was impaired in Dox-treated uPAR-silenced (uPARsi) cells, while there was no difference in recruitment of 19S ATPase subunits. Impaired recruitment of PSMD6 to 26S proteasome complex was further confirmed biochemically in control and uPARsi cells. 26S proteasome was purified from untreated and Dox-stimulated VSMC (Figure S1 A,B). PSMD6 recruitment to the purified proteasome was assessed by western blotting. Together, these data suggest that uPAR may promote proteasome activity in response to genotoxic stress via regulation of 26S proteasome assembly.

**Table 1:** Recruitment of 19S subunits to the proteasome complex calculated as the ratio of normalized intensity of peptide peak to the total input cell extract.

	Enrichment factor		
	Untreated	Dox	uPARsi+Dox
ATPase regulatory subunits			
PSMC2	10	9	11
PSMC1	19	9	11
PSMC4	12	10	10
PSMC6	6.5	7	6
PSMC3	5	6	5.5
PSMC5	8	7	4
Non ATPase regulatorysubunits			
PSMD2	5	4	3.5
PSMD1	10	8.5	6
PSMD3	6	6	4.25
PSMD12	10	9.5	7
PSMD11	5	4.5	4
PSMD6	14	***13***	***8***
PSMD7	22	***18***	***5.5***
PSMD13	11	***10***	***6***
PSMD4	12	8	10
PSMD14	7	8	10
PSMD8	14	15	13

### uPAR mediates DNA single strand breaks signaling and DNA repair

**Figure 1:**
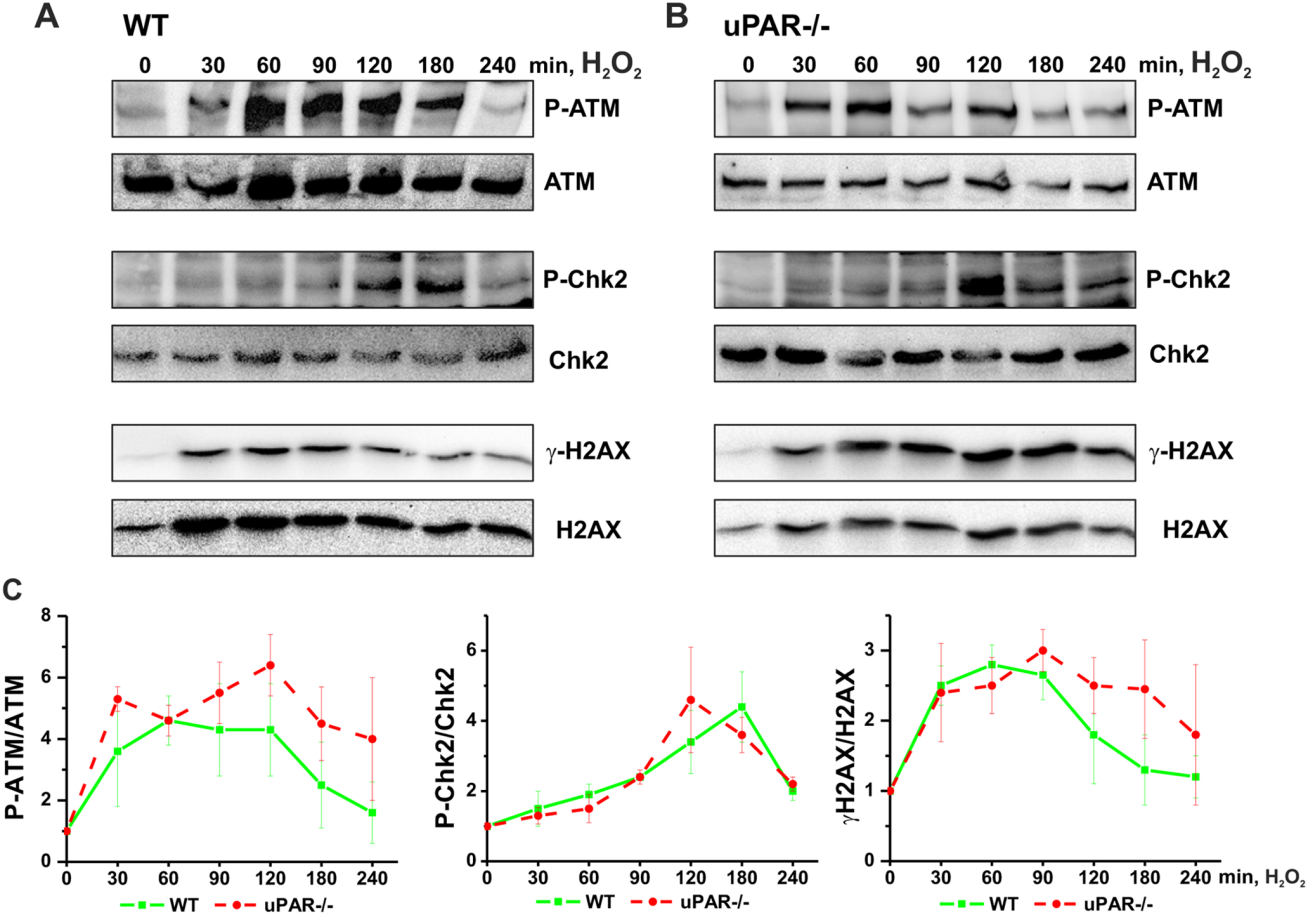
H_2_O_2_ induces DNA DSB signaling in WT and uPAR−/− mouse VSMC. VSMC isolated from WT (A) and uPAR−/− (B) mice were treated with 100 µM H_2_O_2_ for indicated time. Phosphorylation of ATM, Chk-2 and H2AX was assessed by western blotting. C. H_2_O_2_-induced phosphorylation of ATM, Chk-2 and H2AX was quantified from 3 independent experiments. Data are shown as folds of increase relative to unstimulated control and normalized to the total level of corresponding protein.

**Figure 2:**
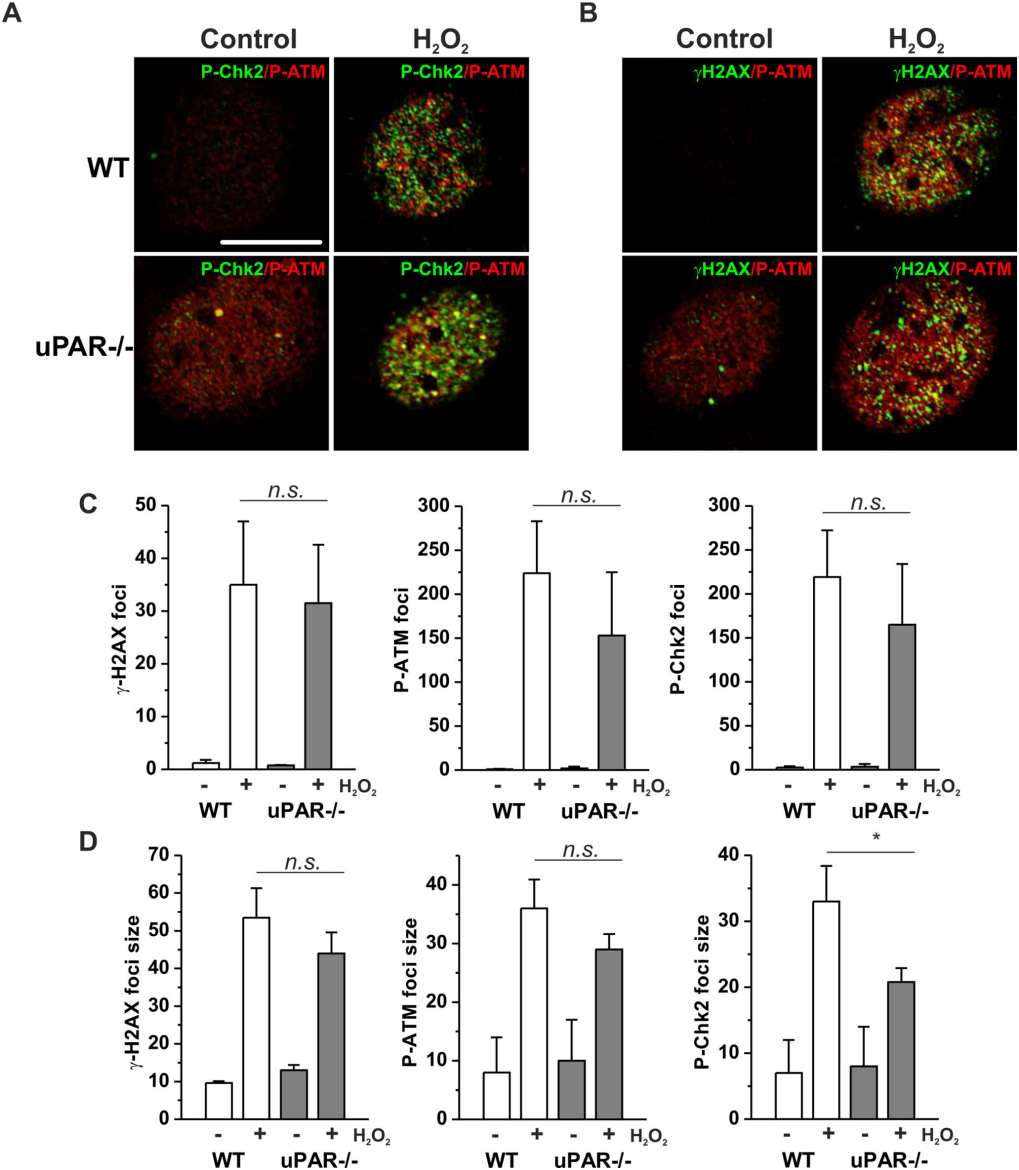
H_2_O_2_ induces DNA damage foci formation in WT and uPAR−/− mouse VSMC. A. WT and uPAR−/− mouse VSMC were treated with H_2_O_2_ for 1 h, then fixed and stained for P-Chk-2 (Alexa 488) and P-ATM (Alexa 594). B. Cells treated as in C were stained for γH2AX (Alexa 488) and P-ATM (Alexa 594). Scale bar 10 µm. C. Quantification of H_2_O_2_-induced DNA damage foci number per cell nucleus was performed using Particle analysis tool of ImageJ. D. Average size of DNA damage foci was calculated using ImageJ.

To get further insights into the role of uPAR in DNA damage-induced regulation of proteasome, we stimulated cells with H_2_O_2_, to avoid interference of Dox fluorescence with immunocytochemical analysis. H_2_O_2_ treatment results in induction of both, DNA single strand breaks (SSB) and DSB. We first assessed H_2_O_2_ -induced DNA DSB signaling in VSMC from WT and uPAR−/− mouse. We observed by western blotting that phosphorylation of histone H2AX (γH2AX), Ataxia telangiectasia mutated kinase (P-ATM) and Checkpoint kinase 2 (P-Chk2) took place in both WT and uPAR−/− cells (Figure 1). We also documented formation of γH2AX/P-ATM and P-Chk2/P-ATM nuclear foci in both WT and uPAR−/− cells at sites of DSB (Figure 2 A,B). Quantification of the immunocytochemical data has not revealed significant difference in the number (Figure 2 C) and intensity (data not shown) of H_2_O_2_ -induced P-ATM, γH2AX and P-Chk2 foci. Average size of P-Chk2 foci was decreased in uPAR−/− VSMC (Figure 2 D). Similar data were obtained using human VSMC nucleofected with control siRNA and uPAR siRNA (data not shown).

**Figure 3:**
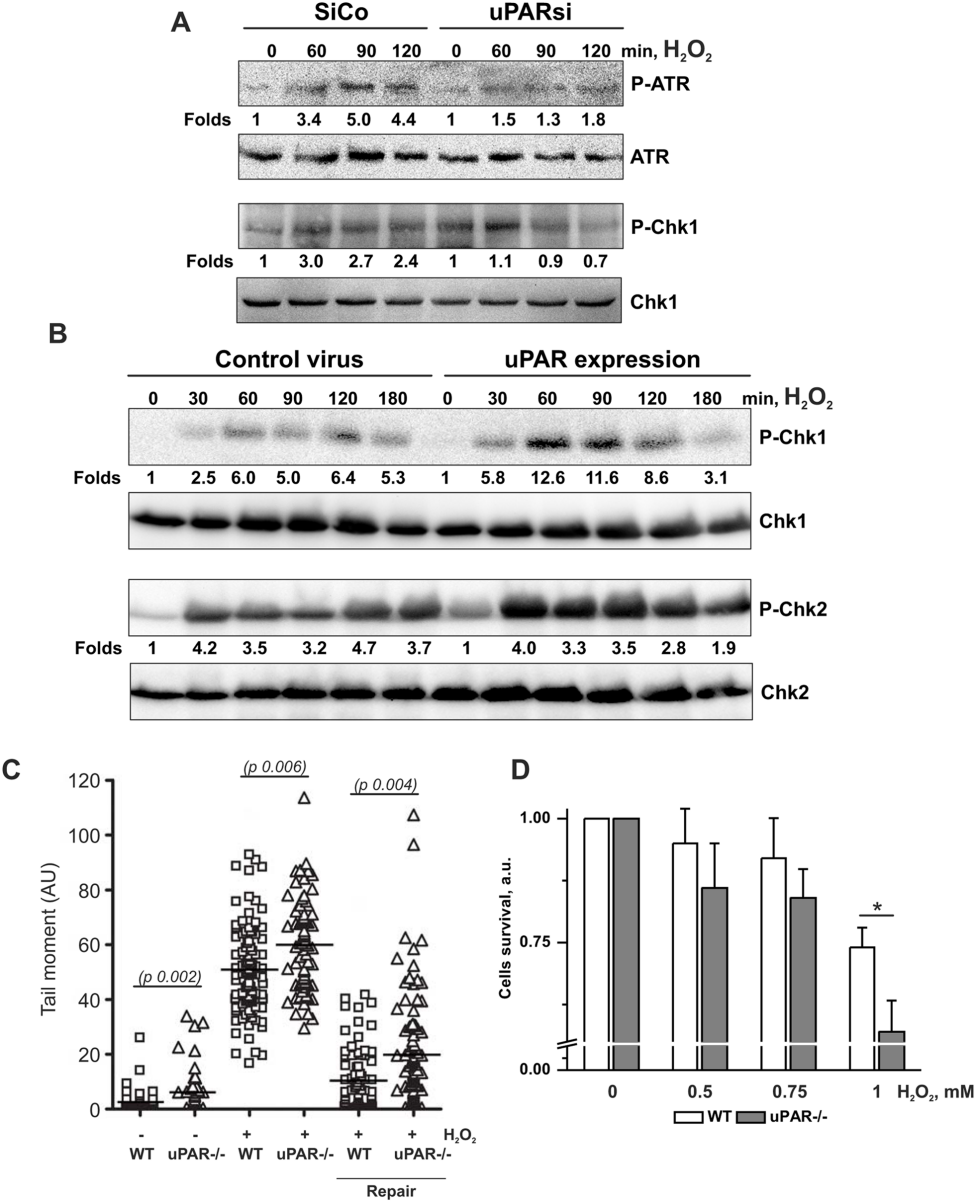
DNA SSB signaling and DNA repair are impaired in uPARsi cells. A. SiCo and uPARsi -nucleofected human VSMC were treated with 100 µM H_2_O_2_ for indicated time points. Phosphorylation of ATR and Chk1 kinase was detected by western blotting. B. HEK 293 cells were infected with control lentivirus or uPAR-FLAG-expressing virus, and stimulated with 100 µM H_2_O_2_ for indicated time points. Phosphorylation of Chk1 and Chk2 kinases was detected by western blotting. C. WT and uPAR−/− mouse VSMC were treated with H_2_O_2_ for 20 min on ice to induce DNA damage. After H_2_O_2_ removal VSMC were allowed to repair DNA for 4 hrs. Comet tails were quantified as described in the Materials and Methods. D. WT and uPAR−/− mouse VSMC were treated with different concentrations of H_2_O_2_ for 20 min on ice to induce DNA damage. The number of viable cells was calculated 24 hrs after DNA damage using 5(6)CFDA as described in Material and methods.

Next, we analyzed SSB-induced signaling in human control siRNA and uPARsi RNA-nucleofected cells. Efficiency of uPAR downregulation using cell nucleofection is shown in Figure S1 C. As shown in Figure 3 A, activation of SSB signaling molecules, namely Ataxia telangiectasia and Rad3-related protein **(**ATR) and Checkpoint kinase 1 (Chk1) kinase was significantly impaired in the absence of uPAR. The level of uPAR expression is shown in Figure S1 D. Similar data was obtained upon uPAR downregulation by means of VSMC lentiviral infection (data not shown). To strengthen these findings pointing to uPAR involvement in activation of SSB repair signaling pathway, we used human embryonic kidney (HEK) 293 cells as a model system. HEK 293 cells do not express endogenous uPAR. We achieved uPAR expression by lentiviral infection and assessed uPAR level by western blotting (Figure S1 C). Control and uPAR-expressing cells were treated with H_2_O_2_ and activation of Chk-1 and Chk-2 kinases were examined. Figure 3 B shows that control cells respond to DNA damage by activation of both, DNA SSB and DSB signaling pathways. However, in uPAR-expressing cells activation of Chk-1 kinase was strongly pronounced. Chk-2 phosphorylation was also more pronounced in uPAR expressing cells. These data suggest that uPAR is especially essential for activation of SSB-induced signaling pathway. Further, we studied whether uPAR-dependent DDR signaling regulation is required for DNA repair and cell survival after DNA damage. Comet assay was performed in H_2_O_2_-treated VSMC. Cells were allowed to repair DNA damage for 3 hrs after peroxide removal. As shown in Figure 3 C, DNA repair was significantly delayed in uPARsi cells. Accordingly, uPAR−/− cells were sensitized to H_2_O_2_ treatment and showed decreased survival in cell viability assay (Figure 3 D).

**Figure 4:**
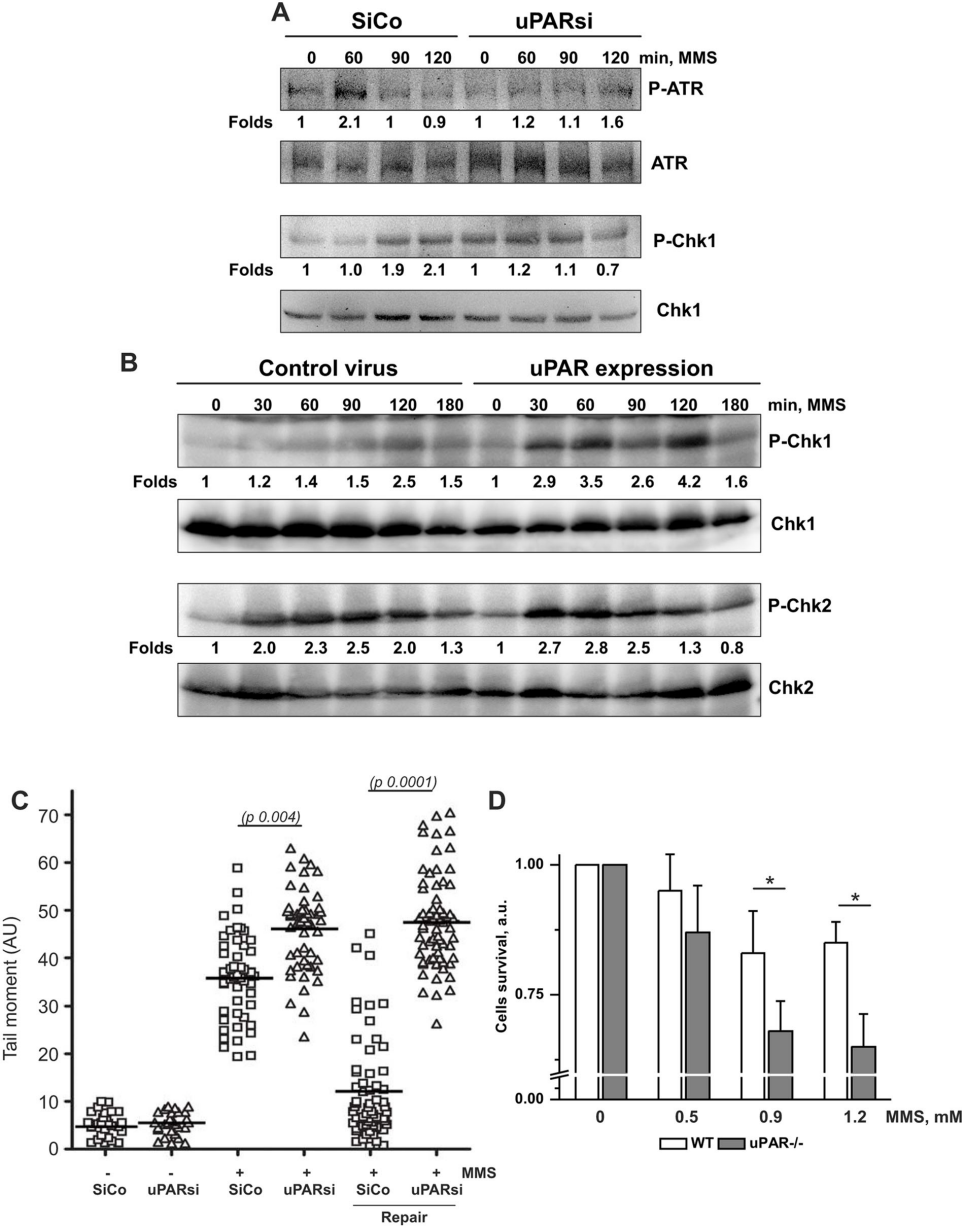
uPAR is essential for MMS-induced DNA SSB signaling and DNA repair. A. Growth arrested SiCo and uPARsi -nucleofected human VSMC were treated with 1.2 mM MMS for indicated time points. Phosphorylation of Chk1 and Chk2 kinases was detected by western blotting. B. HEK 293 cells were infected with control lentivirus or uPAR-FLAG-expressing virus, and stimulated with 1.2 mM MMS for indicated time points. Phosphorylation of Chk1 and Chk2 kinases was detected by western blotting. C. WT and uPAR−/− mouse VSMC were treated with MMS for 20 min on ice to induce DNA damage. After H_2_O_2_ removal VSMC were allowed to repair DNA for 4 hrs. Comet tails were quantified as described in the Materials and Methods. D. WT and uPAR−/− mouse VSMC were treated with different concentrations of MMS for 20 min to induce DNA damage. The number of viable cells was calculated 24 hrs after DNA damage using 5(6)CFDA as described in Material and methods.

Our data point to the involvement of uPAR in DNA SSB-induced signaling pathway. H_2_O_2_ treatment results, however, in induction of both, DNA SSB and DSB that cannot be distinguished by comet assay. Therefore, we next treated cells with the alkylating agent MMS. In growth-arrested cells treated with low MMS concentrations, SSB are produced during Base Excision Repair (BER). In agreement with the above data, activation of Chk1 kinase after MMS treatment was significantly impaired in uPAR-deficient cells (Figure 4 A). Western blotting showing uPAR expression is shown in Figure S1 D. Accordingly, uPAR-expressing HEK cells showed stronger activation of Chk1 than control cells (Figure 4 B). Comet assay was further performed to estimate the SSB repair in uPAR deficient cells. The data showed that DNA repair was delayed in uPAR-deficient cells (Figure 4 C) and cell survival was decreased (Figure 4 D). In order to test whether the observed mechanism of uPAR-dependent regulation of SSB signaling is cell type specific, comet assay was performed in MDA-MB 231 cancer cell line. Expression of uPAR was downregulated by means of siRNA and cell nucleofection. As shown in Figure S1 D, uPARsi cancer cells also revealed delayed DNA repair in response to MMS treatment.

Together, these data provide evidence for necessity of uPAR in induction of DNA SSB-dependent signaling and DNA repair.

### uPAR regulates PSMD6 nuclear translocation and assembly of 26S proteasome complex

**Figure 5:**
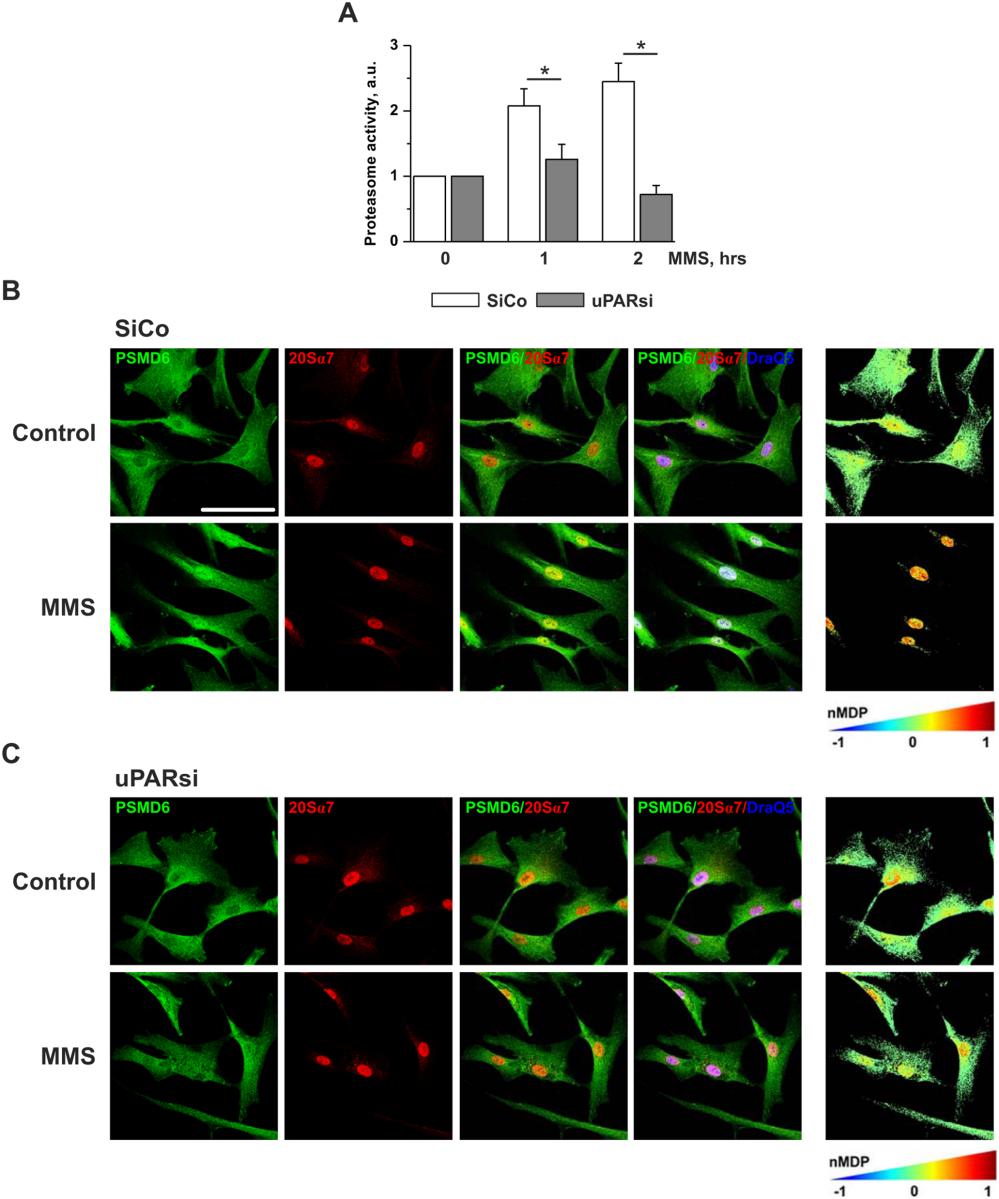
MMS-induced increase of proteasome activity and PSMD6 nuclear import are abrogated in uPARsi VSMC. A. SiCo and uPARsi human VSMC were treated with 1.2(B) and uPARsi (C) VSMC were treated with 1.2 mM MMS for 1 h, then fixed and stained for PSMD6 (Alexa 488) and 20Sα7 subunit (Alexa 594). DraQ 5 was used as nuclear stain. The right panels in B, C show colocalization of PSMD6 and 20Sα7 indicated by color coding. The colormap was created using colocalization colormap plugin of ImageJ software. Scale bar 100 µm.

**Figure 6:**
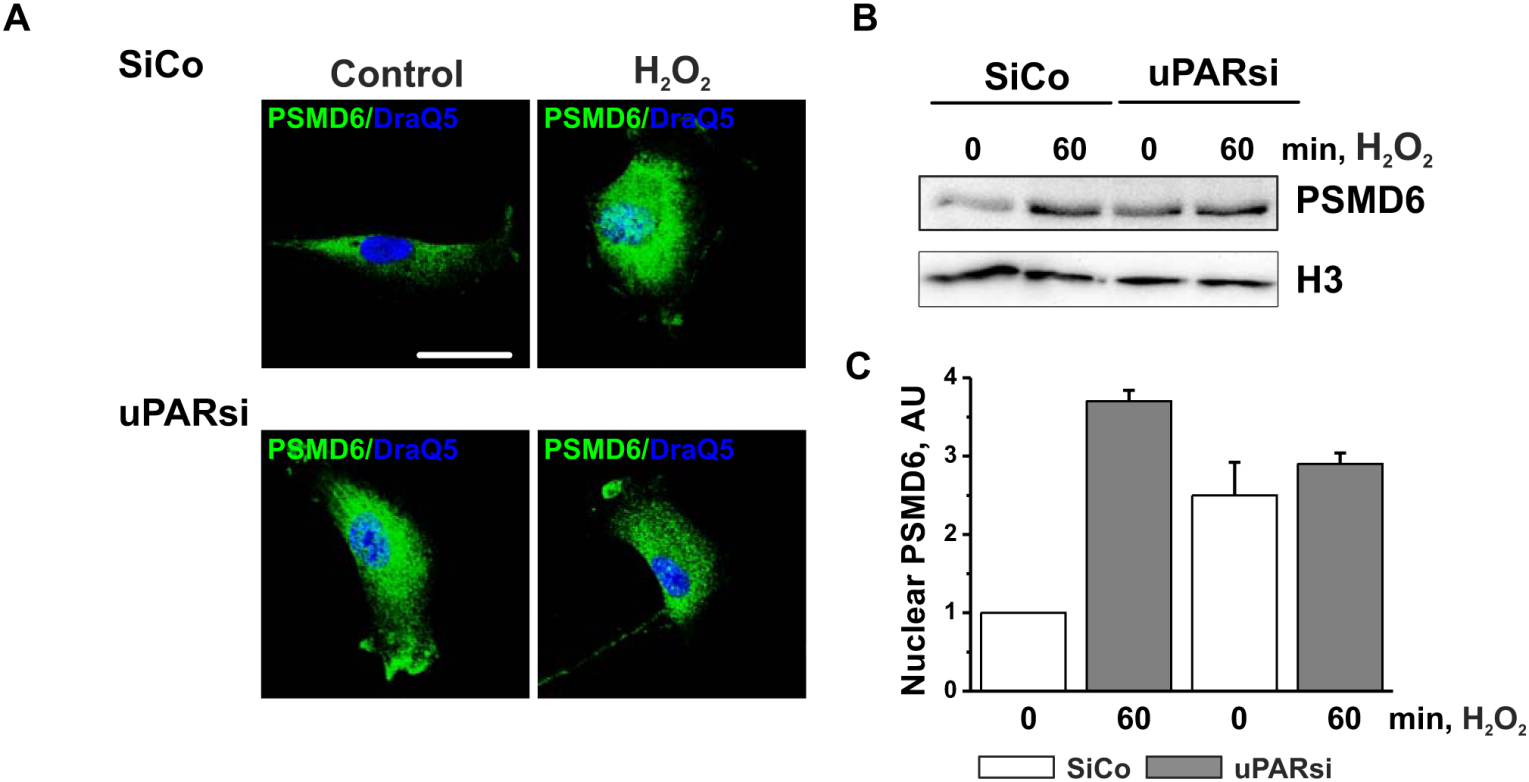
H_2_O_2_-induced PSMD6 nuclear import is impaired in the absence of uPAR. A. SiCo and uPARsi -nucleofected human VSMC were treated with 100 µM H_2_O_2_ for 1 h at 37°C. Then cells were fixed and stained for PSMD6 (Alexa 488) DraQ5 was used as nuclear stain. B. Human VSMC were treated as in A and subcellular fractionation was performed. PSMD6 content in nuclear fraction was assessed by western blotting. Histon H3 was used as loading control. Scale bar 100 µm. C. H_2_O_2_ -induced nuclear import of PSMD6 was quantified from 3 independent experiments.

Further we examined whether uPAR-dependent propagation of the MMS-induced DNA damage might be mediated by proteasome. Similar to Dox treatment, cell stimulation with MMS resulted in upregulation of proteasome activity. This effect was abrogated in uPARsi cells (Figure 5 A). Based on our MS data on impaired proteasome assembly in uPAR deficient cells after Dox treatment, we examined control and MMS-treated cells for different 19S regulatory subunits by immunocytochemistry. Among the analyzed subunits, PSMD6 showed very pronounced subcellular redistribution after induction of DNA damage, whereas others did not alter their subcellular localization (Figure S2). In resting cells, PSMD6 was localized primarily in cytoplasm and was recruited to the cell nucleus after treatment with MMS (Figure 5 B). Interestingly, DNA damage-induced nuclear translocation of PSMD6 was abolished by downregulation of uPAR expression (Figure 5 C). In nucleus, significant colocalization of PSMD6 and 20S core particle was observed suggesting the increased assembly of the 26S proteasome after DNA damage (Figure 5 B). Analysis of PSMD6/20Sα7 subunit colocalization performed using colocalization colormap plugin of ImageJ software revealed strong nuclear 26S proteasome assembly after MMS stimulation (Figure 5 B, right panel). By contrast, MMS treatment did not cause redistribution of PSMD6/20Sα7 colocalization in uPARsi cells (Figure 5 C, right panel). Similar uPAR-dependent DNA damage-induced PSMD6 nuclear import was also observed in H_2_O_2_ treated cells as assessed by immunocytochemistry and cell fractionation (Figure 6 A–C) and in MDA-MB 231 cancer cells (Figure S3).

**Figure 7:**
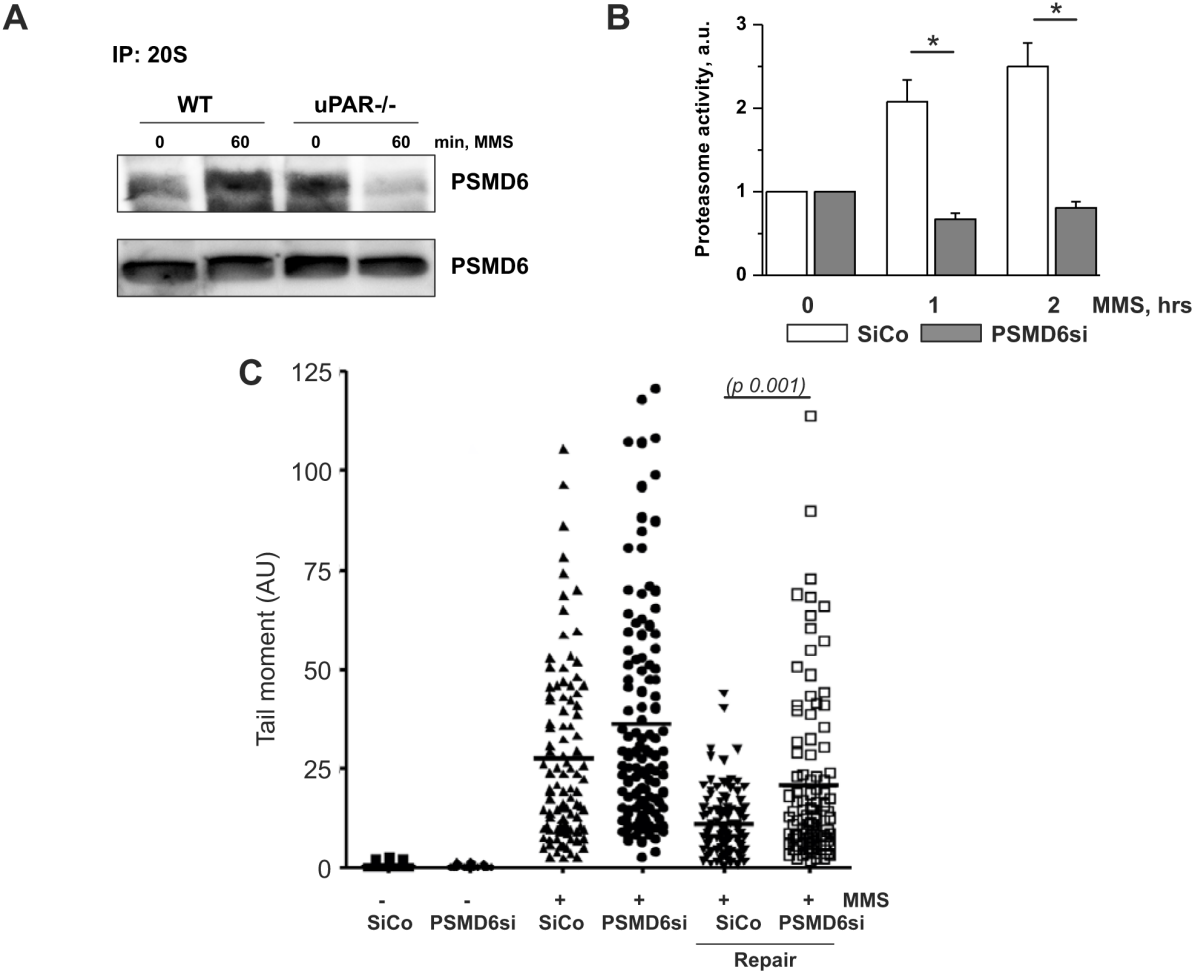
uPAR regulates PSMD6 recruitment to 26S proteasome and its activity. A. SiCo and uPARsi human VSMC were treated with 1.2

Next, we examined by means of immunoprecipitation whether PSMD6 is recruited to 26S proteasome complex. We used 20S core particle antibodies and detected PSMD6 in the immunoprecipitates. Indeed, our data showed that MMS treatment leads to recruitment of PSMD6 to proteasome (Figure 7 A). In functional experimental settings, silencing of PSMD6 abolished MMS-induced upregulation of proteasome activity and significantly delayed DNA repair as assessed by the comet assay (Figure 7 B,C). Efficiency of PSMD6 silencing is shown in Figure S1 C.

Together, our data show that uPAR regulates DNA damage-induced proteasome activity via nuclear translocation of PSMD6 and nuclear assembly of 26S proteasome complex.

### uPAR controls PSMD6 serine phosphorylation

It was an intriguing question, how uPAR may regulate nuclear import of PSMD6 in response to DNA damage. We were interested to know whether PSMD6 redistribution and functions might be regulated by its posttranslational modifications. Several kinds of posttranslational modifications have been demonstrated for proteasome subunits and implicated in their functions. In particular, poly(ADP)ribosylation (PARylation) [19], [20], serine phosphorylation [21], acetylation of some 19S subunits, but not PSMD6 [21], ubiquitination of 20S proteasome subunits were shown [22].

**Figure 8:**
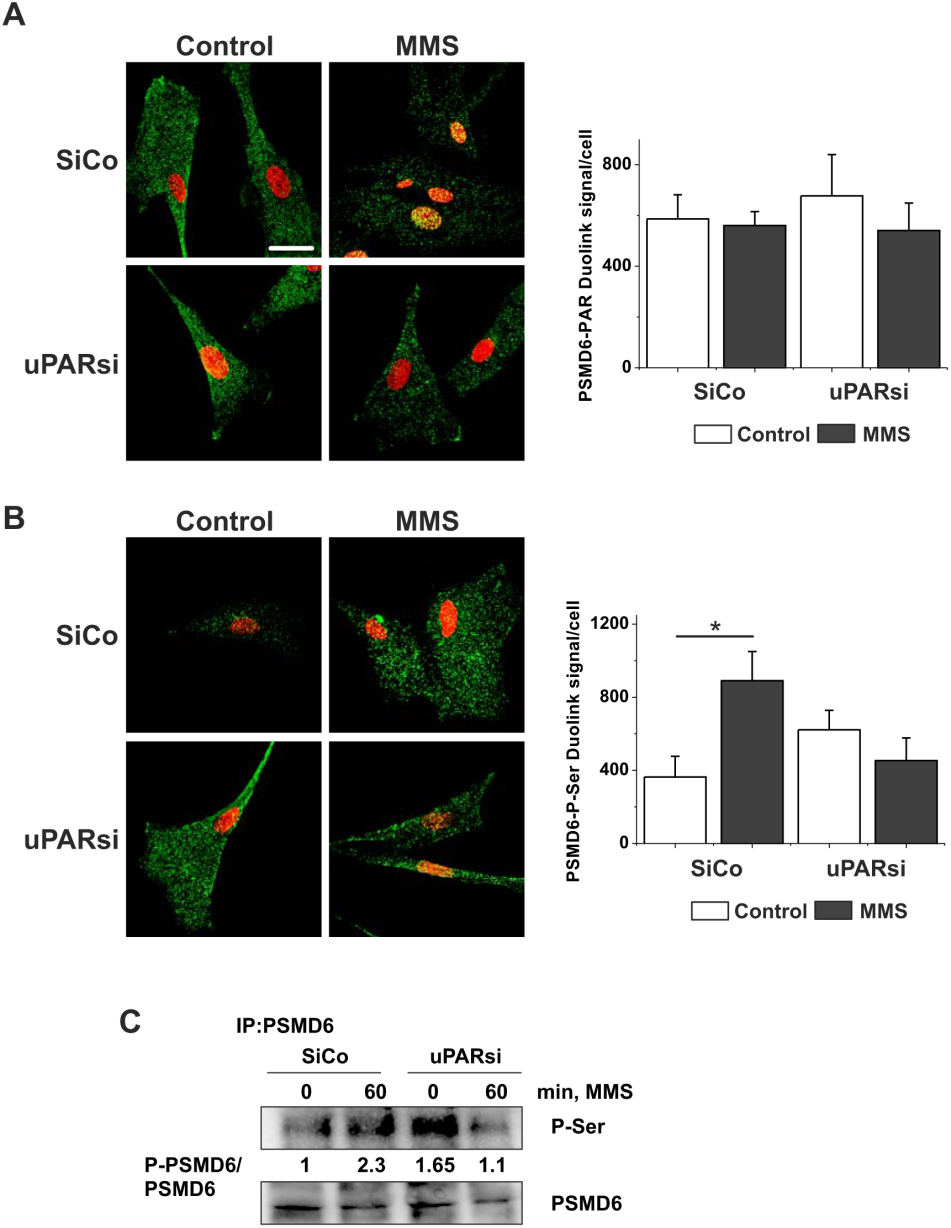
uPAR is required for MMS-induced PSMD6 phosphorylation. A. SiCo and uPARsi human VSMC were treated with 1.2(A) and serine phosphorylation (B) was assessed using Duolink proximity ligation assay. The right panels show the number of Duolink PLA signals per cell quantified using ImageJ software. DraQ5 was used as nuclear stain. C. Serine phosphorylation of PSMD6 was assessed by immunoprecipitation. PSMD6 from lysates of control and MMS-stimulated cells was immunoprecipitated. P-Ser was detected by western blotting. The lower panel shows loading controls.

We addressed first PSMD6 PARylation using Duolink proximity ligation assay (PLA). It was shown previously that PARylation of nuclear proteasome by PARP-1 increases proteasome activity and facilitates degradation of oxidatively damaged histones [19]. As shown in Figure 8 A, in unstimulated cells PARylated PSMD6 was present in the cytoplasm. Though the level of PSMD6 PARylation was not affected by MMS treatment, MMS induced accumulation of modified PSMD6 in the cell nucleus. The level of PSMD6 PARylation in uPARsi cells was not changed after MMS treatment. However, there was no nuclear translocation of modified PSMD6. In further experiments neither changes in PSMD6 ubiquitination nor in PSMD6 acetylation were found in response to MMS treatment or uPAR silencing (data not shown).

Finally we asked whether phosphorylation of PSMD6 might be under control of uPAR and DNA repair machinery. We relied again on PLA experimental approach to detect the degree and localization of PSMD6 phosphorylation. The number of PLA signals per cell was quantified using ImageJ software. As shown in Figure 8 B, serine phosphorylation of PSMD6 was upregulated after MMS treatment. Though basal level of phosphorylation was present in uPARsi VSMC, MMS-induced increase in phosphorylation was not observed in these cells. These data were further confirmed by immunoprecipitation experiments (Figure 8 C).

## Discussion

The DDR network plays a cardinal role in the maintenance of genome integrity and is, as one of the key cellular mechanisms, a subject of intensive research. Multiple studies have addressed the role of proteasome in DNA damage sensing and repair, and postulated that functional proteasome is essential for effective repair process. These observations promoted application of proteasome inhibitors along with the genotoxic drugs in cancer treatment to prevent DNA repair and increase therapeutic efficiency [7]. However, data on the role of the proteasome in DDR machinery are controversial and mechanisms underlying regulation of proteasome assembly and activity remain less explored.

Our data revealed a new pathway in these mechanisms linking regulation of proteasome assembly and activity to the plasminogen activation system, in particular to the multifunctional receptor uPAR. We show that uPAR serves as a cellular sensor for DNA-damaging signal and that loss of uPAR sensitizes cells to DNA damage and retards DNA repair. We provide evidence that uPAR mediates specifically DNA SSB signaling and DNA repair. We identified PSMD6 as a proteasome subunit regulated by uPAR. DNA damage induces redistribution of PSMD6 to the nucleus and results in increased proteasome activity in an uPAR-dependent fashion.

The uPA/uPAR is a surprisingly multifaceted system upregulated upon numerous diseases, primarily those related to inflammation, tissue remodeling and cancer [14], [15], [17]. At the level of cellular functions determining the cell fate in response to microenvironment, uPAR-directed signaling is believed to regulate physiological and pathophysiological conditions requiring changes in cell proliferation, migration, adhesion, and survival [15]. uPAR realizes two important cellular functions providing regulation of extracellular proteolytic cascade and serving as a signaling receptor to promote changes in cell functional behavior [16]. As a GPI-anchored receptor lacking transmembrane and intracellular domains, uPAR associates with transmembrane proteins, such as integrins, tyrosine kinase receptors and others, to initiate multiple signal transduction pathways [17]. Due to its multifunctional properties uPAR offers many opportunities to be utilized as a target for specific therapies in diverse human diseases. However, none of the earlier studies addressed possible involvement of uPAR in response to DNA damage. One recent study related to this issue has shown that transcriptional silencing of metalloproteinase 9 in combination with uPAR/cathepsin B affected DSB repair machinery in human glioma in vitro and in vivo [23]. One further report from the same group suggested that inhibition of uPAR together with cathepsin B might be used in radiation therapy to target glioma-initiating cells [24]. However, the impact of uPAR on DDR-related mechanisms independently of cathepsin B was not explored in those studies.

We provide clear evidence for requirement of uPAR for cellular response to DNA damage and repair. However, the question how DNA damaging signals may induce uPAR activation remains unclear and requires further research. Most of the studies addressing DDR are focused on the coordinated mechanisms by which DDR proteins orchestrate at the site of DNA damage. Cellular effects of DNA-damaging agents are not limited, however, to induction of DNA lesions. Mitochondrial release of reactive oxygen species has proven to be powerful participant of DNA damage sensing and repair, in particular, after MMS treatment [25]. ROS inhibit protein phosphatases and thus may increase phosphorylation of cytoplasmic proteins and plasma membrane receptors, for example ErbB receptor family [26]. Activation of membrane receptors might be essential for DNA repair. Thus, epidermal growth factor receptor (EGFR) can modulate repair process via nuclear translocation and association with the catalytic subunit of DNA-dependent protein kinase (DNA-PK). Furthermore, direct and indirect posttranslational modifications of cytoplasmic proteins by DNA damaging drugs have also been reported [27], [28]. Multifunctionality of uPAR and its high ability for various co-receptors binding offer multiple possibilities for ROS-mediated activation of receptors and downstream signaling. Another potential mechanism for uPAR-mediated proteasome assembly might be suggested based on the study by Asuthkar et al. [18]. It was reported that heat-shock protein 90 (HSP90) mediates uPAR interaction with β-catenin after cells exposure to ionizing radiation. Since HSP90 chaperons the assembly of 26S proteasome [29], it is an attractive model of direct uPAR interference with proteasome assembly.

Data from our MS analysis suggest that uPAR serves for induction of changes in proteasome 19S lid complex assembly in response to DNA damage signals, such as Dox, H_2_O_2_ and MMS. We found that loss of uPAR resulted in deregulation of the 26S proteasome subunit PSMD6, which is an integral component of DDR. Our experiments on PSMD6 posttranslational modifications suggest that PSMD6 phosphorylation might influence mechanisms of its nuclear import. PSMD6 PARylation, ubiquitination and acetylation are most likely not required for its nuclear import. Mechanisms regulating nuclear import of PSMD6, identification of kinase and phosphatase regulating the level of PSMD6 phosphorylation, along with mechanisms of PSMD6 nuclear import remain intriguing questions for further research. One further interesting issue for future studies is a 26S proteasome-independent role, which nuclear PSMD6 and/or 19S may have in DDR signaling and repair.

Our study points to a critical role of DDR in vascular cells not only upon anti-cancer treatments but in cardiovascular diseases as well. Increasing body of evidence suggests that vascular cells represent important target for cytotoxic therapies. Thus, aortic VSMC accumulate Dox several hours after application [30]. Large patient cohorts studies confirmed increased incidence of cardiovascular events after anti-cancer treatment [11]. Extensive DNA damage originating from altered oxidative status in the atherosclerotic plaque area has been reported. Further studies suggest a causative role for DNA damage in atherosclerosis, rather than being just its passive consequence. However, response of vascular cells to DNA damage and cellular and functional consequences in the context of vascular wall has been largely not addressed. Our findings strengthen a novel function for uPAR documented in our recent reports and classify uPAR as an important regulator of intracellular proteolysis controlling ubiquitination and proteasomal degradation of proteins determining fate and functional behavior of vascular cells in response to DNA damage. Identification of other UPS components beyond PSMD6 that might be regulated by uPAR is a question of great importance that may have an impact in the development of new therapeutic strategies aiming at targeting uPAR.

## Materials and Methods

### Ethics Statement

Isolation of cells from mouse tissues was carried out according to the European Commission guidelines and was approved by the ethics committee of Hannover Medical School.

### Mass spectrometry analysis of proteasome assembly

Proteasome complex and its interacting proteins were purified from Dox-treated control and uPARsi cells using proteasome purification kit (Enzo Life Sciences). Isolated proteins were subjected to ESI-LTQ Orbitrap mass spectrometer (Thermo Fisher Scientific) for quantitative analysis. Enrichment factors were calculated for each individual peptide as the ratio of normalized intensity of peptide peak to the total input cell extract.

### Cell culture, cell nucleofection, infection, and treatment with H_2_O_2_ and MMS

Human primary coronary artery VSMC were purchased from CellSystems and cultivated as recommended by the supplier. Aortic VSMC were isolated from male uPAR−/− mice and uPAR+/+ (wild type) mice (all on C57/BL6 background, age 10–12 weeks). Animals were euthanized by intravenous injection of 200 µl 2% avertin solution. The aortas were dissected, cut into 1–2 mm pieces and subjected to enzymatic digestion as described [31]. Mouse VSMC were cultivated in DMEM (Dulbecco’s modified Eagle’s medium) supplemented with 10% (v/v) fetal bovine serum. MDA-MB-231 human breast cancer cell line (American Type Culture Collection, Rockville, MD) were cultured in Dulbecco’s modified Eagle’s medium (DMEM, Lonza) supplemented with 10% fetal bovine serum (PromoCell GmbH).

DDR pathway activation was induced by cell treatment with 100 µM H_2_O_2_ at 37°C. For Comet assay, cells were treated with 5 mM H_2_O_2_ for 20 min on ice. MMS was used in final concentration 1.2 mM. For Comet assay, cells were treated with 0.64 µM MMS. H_2_O_2_ and MMS were purchased from Sigma.

Small interfering RNAs (siRNAs) for downregulation of uPAR expression and control silencing RNA were obtained from Santa Cruz Biotechnology and were transfected to the human VSMC using Amaxa Nucleofector (Lonza). Basic Primary Smooth Muscle cell nucleofector kit (Lonza) was used according to the manufacturer’s instructions. Cell Line Nucleofector Kit V (Lonza) was used for MDA-MB-231 nucleofection. Lentivirus for downregulation of uPAR expression was prepared as reported previously [32]. uPAR overexpressing lentivirus was used as reported [33]. shRNA for PSMD6 (TRCN0000143904) was obtained from Sigma’s MISSION shRNA library.

### Immunostaining

Cells grown on coverslips were fixed by addition of 10% formaldehyde to the final concentration of 2%, permeabilized in 0.1% Triton X-100 for 10 min and blocked with 3% (w/v) BSA/PBS at 4°C overnight. Cells were labeled with primary and corresponding Alexa Fluor 488- or Alexa Fluor 594-conjugated secondary antibody (Invitrogen) for 1 h at room temperature. Cells were then mounted with Aqua-Poly-Mount mounting medium (Polysciences) and analyzed on a Leica TCS-SP2 AOBS confocal microscope. For immunostaining of mice Aortic VSMC, cells were incubated with 5% mouse serum in PBS followed by 1 h incubation with 5% normal goat serum.

Duolink In Situ Proximity Ligation Assay (PLA) Probes (anti-rabbit PLA probe PLUS, anti-mouse PLA probe MINUS, and Duolink In Situ Detection Reagent Green) were purchased from Sigma-Aldrich and used accordingly to the manufacturer’s instructions. The number of PLA signals was quantified using ImageJ software.

### Preparation of cell lysates, Immunoprecipitation and Western Blotting

Cultured cells were lysed in RIPA buffer containing 1 mM PMSF, 1 µg/ml aprotinin, 1 µg/ml leupeptin, 1 mM Na_3_VO_4_, 1 mM NaF and incubated for 10 min at 4°C. For whole cell lysate preparation lysates were subjected to sonication. The lysates were centrifuged at 10,000 rpm for 10 min. For immunoprecipitation 600 µg total cell lysate with 4 µg of specific antibodies was used. After 3 hours immunocomplexes were precipitated with A/G PLUS-agarose beads. Precipitates were washed 3 times in PBS buffer containing protease inhibitors and subjected to SDS-electrophoresis.

Isolation of cytosolic and nuclear fractions was performed as described [34].

Antibodies against P-Chk-1, Chk-1, P-Chk2, γH2AX, H2AX, Phospho-ATM, ATM, Phospho-ATR, ATR, Histone H3 were from Cell Signaling. Antibodies for Chk-2 and P-Chk-2 (sc-16297-R) were from Santa Cruz. Anti-uPAR monoclonal antibody was from R&D Systems. Antibodies against 19S proteasome subunits were from Enzo Life Sciences. Anti-tubulin antibody was from BD Pharmingen. Alexa Fluor 488-conjugated chicken anti-rabbit antibody and Alexa Fluor 594-conjugated donkey anti-mouse antibody were from Life Technologies.

Western blotting images were acquired using VersaDoc Imaging system (Bio-Rad) and quantified using QuantityOne software (Bio-Rad). Expression of phosphorylated proteins was normalized to the level of total protein expression.

### Proteasome activity assay

Total proteasomal activity in cell lysates was measured using the 20S proteasomal assay kit (Cayman Chemical Company, Ann Arbor, Mich., USA) as described by the manufacturer. In brief, VSMC were treated with different concentrations of MMS. The cell lysates were incubated with 10 µM substrate (SUC-LLVY-AMC) for 1 h at 37°C, the fluorescence was read using a Magellan GENIOUS (Tecan, Männedorf, Switzerland) at 360 nm (excitation) and 480 nm (emission). The enzymatic activity was normalized to the protein concentration. The results are reported as means ± SD.

### Comet assay

Comet assay was performed as described [35] with some modifications. Briefly, cells were stimulated with H_2_O_2_ or MMS for 1 h, trypsinised and counted. Approximately 10,000 cells were mixed with 1% low melting agarose and spread on normal agarose pre-coated glass slides. After the agarose solidification for 30 min at 4°C, slides were incubated in lysis buffer (1% Triton X-100 in 10 mM Tris, 100 mM EDTA) for 2 h, at 4°C. The slides were incubated in alkaline running buffer (10 N NaOH, 200 mM EDTA, pH>13) for 20 min, before performing electrophoresis for 20 min at 300 mA, 25 V. After electrophoresis the slides were 2×5 min incubated with neutralization buffer (0.4 M Tris, pH-7,5), placed in cold 100% ethanol for 5 min and dried overnight at 4°C. Staining was performed with vista green dye (Cell Biolabs) and comets were observed under a fluorescence microscope. Analysis of comet tails was performed by ImageJ and CASP (CaspLab) Softwares. 50–100 cells were used for quantification of comet tails.

### Statistical analysis

All experiments were performed at least three times. Statistical significance analysis (*P<*0.05) was performed using a Student’s *t* test. “*” represents statistically significant differences at P<0.*05*.

## Supporting Information

Figure S1 A. PSMD6 recruitment to 26S proteasome. Proteasomes were isolated using proteasome purification kit (Enzo Life Sciences). PSMD6 was detected by western blotting. 20S a7 subunit was used as loading control. B. Quantification of western blot shown in A. C. uPAR expression downregulation in human VSMC by means of cell nucleofection with scrambled (SiCo) and uPARsi RNA(left); uPAR overexpression in HEK 293 cells infected with control and uPAR-expressing lentivirus (middle); PSMD6 downregulation in VSMC (right). D. uPAR expression in SiCo and uPARsi VSMC treated with peroxide (upper panels) and MMS (lower panels). E. MMS-induced DNA damage repair in SiCo and uPARsi MDA-MB 231 cells assessed by comet assay.
(TIF)

Figure S2 SiCo and uPAR si VSMC were treated with MMS for 1 h, then fixed and stained for 19S regulatory subunits. Sale bar 10 µm. PSMD7 distribution is shown in mouse VSMC.
(TIF)

Figure S3 MMS-induced PSMD6 redistribution and colocalization with 20Sα7 subunit in SiCo and uPARsi MDA-MB 231. The right panels show colocalization of PSMD6 and 20Sα7 indicated by color coding. The colormap was created using colocalization colormap plugin of ImageJ software. Scale bar 100 µm.
(TIF)
