# Supplementary figures and images for: Loss of Urokinase Receptor Sensitizes Cells to DNA Damage and Delays DNA Repair
Source: PLoS One. 2014 Jul 2;9(7):e101529. doi: 10.1371/journal.pone.0101529 (PMC4079571; doi:10.1371/journal.pone.0101529)

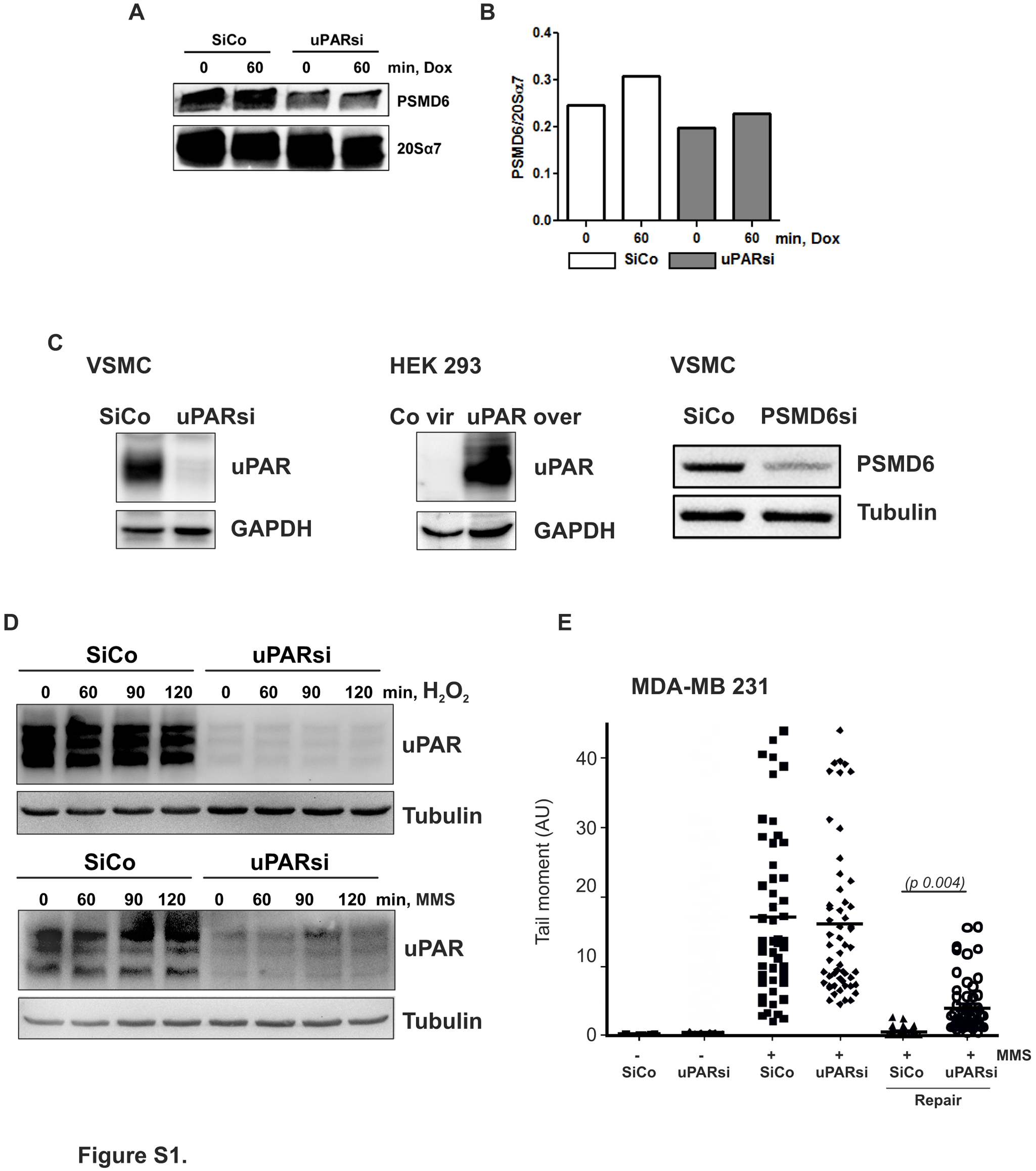

Supplement: Figure S1 — A. PSMD6 recruitment to 26S proteasome. Proteasomes were isolated using proteasome purification kit (Enzo Life Sciences). PSMD6 was detected by western blotting. 20S a7 subunit was used as loading control. B. Quantification of western blot shown in A. C. uPAR expression downregulation in human VSMC by means of cell nucleofection with scrambled (SiCo) and uPARsi RNA(left); uPAR overexpression in HEK 293 cells infected with control and uPAR-expressing lentivirus (middle); PSMD6 downregulation in VSMC (right). D. uPAR expression in SiCo and uPARsi VSMC treated with peroxide (upper panels) and MMS (lower panels). E. MMS-induced DNA damage repair in SiCo and uPARsi MDA-MB 231 cells assessed by comet assay. (TIF) [file pone.0101529.s001.tif]

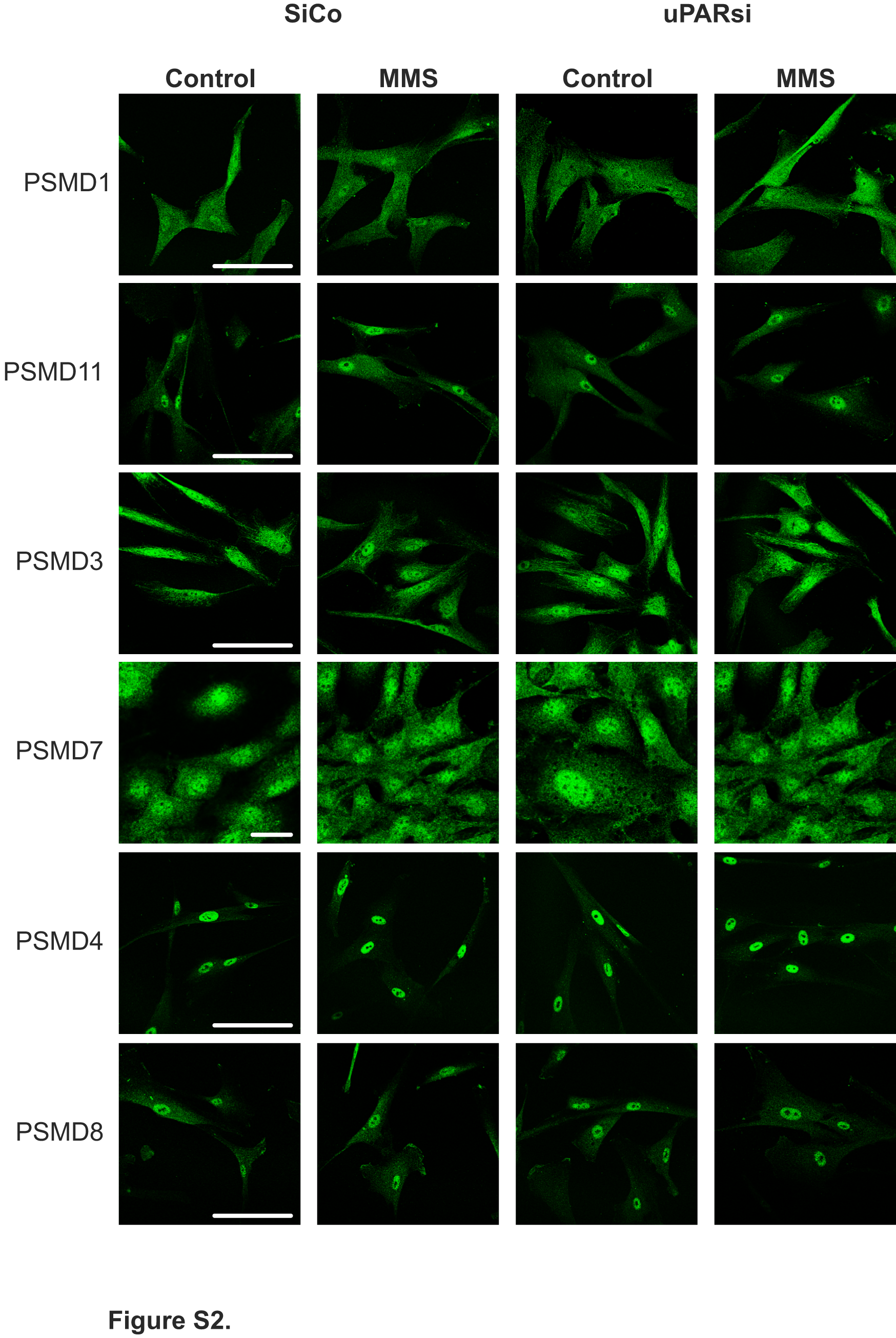

Supplement: Figure S2 — SiCo and uPAR si VSMC were treated with MMS for 1 h, then fixed and stained for 19S regulatory subunits. Sale bar 10 µm. PSMD7 distribution is shown in mouse VSMC. (TIF) [file pone.0101529.s002.tif]

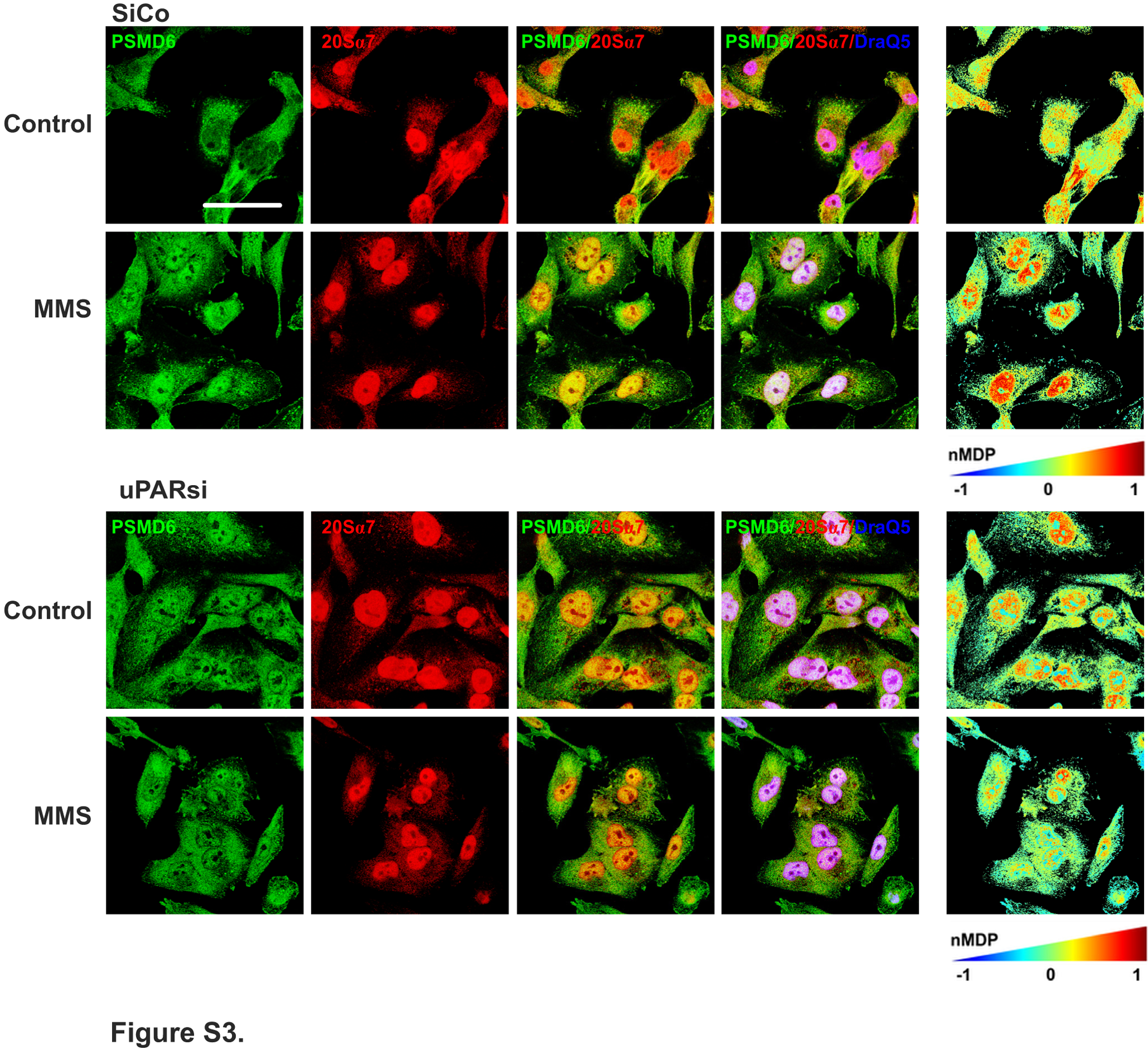

Supplement: Figure S3 — MMS-induced PSMD6 redistribution and colocalization with 20Sα7 subunit in SiCo and uPARsi MDA-MB 231. The right panels show colocalization of PSMD6 and 20Sα7 indicated by color coding. The colormap was created using colocalization colormap plugin of ImageJ software. Scale bar 100 µm. (TIF) [file pone.0101529.s003.tif]
